# Measuring dynamic emotional experiences in response to media stimuli

**DOI:** 10.3389/fpsyg.2024.1436918

**Published:** 2024-12-17

**Authors:** Julia R. Winkler, Markus Appel

**Affiliations:** Psychology of Communication and New Media, Human-Computer-Media Institute, University of Würzburg, Würzburg, Germany

**Keywords:** emotional dynamics, continuous measurement of emotions, change processes, media reception, emotion measurement

## Abstract

Communication research has long recognized the dynamic nature of most media stimuli and the corresponding dynamic emotional processing implied on the side of the audience. Capturing and analyzing these emotional dynamics during media reception is imperative for advancing our understanding of media processing and effects, but is not common practice in most communication research. This article highlights several methodological approaches to measuring the physiological, behavioral, and experiential components of emotions during media exposure: Electrodermal activity, automated facial expression analysis, continuous response measurement, and self-probed emotional retrospections. We discuss these methods in terms of what they measure, their practical application to different research contexts, and aspects of data-analysis. We further highlight ways to adapt and apply these methods to advance research on hot topics in communication science, psychology, and related fields and provide recommendations for scholars who wish to integrate continuous measures of emotional responses into their research.

## Measuring dynamic emotional experiences in response to media stimuli

Most media stimuli are complex and dynamic. These dynamic properties of media such as movies and TV shows, books, music, games, social media feeds, and journalistic content have been widely described both theoretically and empirically (Boyd et al., 2020; Green and Appel, 2024; Ip, 2011; Orjesek et al., 2022; Wise et al., 2010). Important theories of media psychology, such as affective disposition theory (Raney, 2006, 2017; Zillmann and Cantor, 1977), excitation transfer theory (Zillmann, 1971, 2006), and the limited capacity model of motivated mediated message processing (LC4MP) (Lang, 2000, 2006; Keene and Lang, 2016) connect the content features of media stimuli to dynamic emotional responses and/or postulate effects of emotional change (see Nabi, 2015; Nabi and Green, 2015). For empirical research dealing with media processing, it is therefore important, if not essential, to capture the dynamic emotional responses to media stimuli that are key elements of pertinent theoretical approaches. Today, however, only few empirical research projects involve measures of dynamic emotional audience responses during media reception (Schmälzle and Huskey, 2023; Walter et al., 2018).

The aim of this paper is to address this major shortcoming in the literature on media responses and effects. We review classic and novel methodological approaches to measuring dynamic emotional responses. A range of methods are available to do so, all of which come with unique challenges, advantages, and limitations and are suitable for different research contexts. This review aims to help novice and seasoned researchers alike who are new to the field of emotion measurement and interested in emotional processes during media reception, by providing an overview of the methodological toolkit available, guiding them toward relevant in-depth resources, and supporting informed decision-making.

We cover different methodological approaches that are compatible with different media formats (e.g., visual, audio or audiovisual media) and allow the assessment of physiological, behavioral/expressive, and/or subjective experience components of emotions. Next to well-established methods (e.g., psychophysiological measures), we discuss less common but promising ways to capture dynamic emotional responses, providing a brief overview of the general procedure, suitable application contexts, advantages and limitations, and data-analytic strategies. Furthermore, we discuss how these methods can be applied to investigate relevant research questions in key domains of media psychology, in particular, research on narrative effects, social media, and virtual reality. We conclude our article with recommendations for scholars intending to incorporate measures of emotion into their research.

## Theoretical and methodological considerations of dynamic emotion measurement

**Figure 1:**
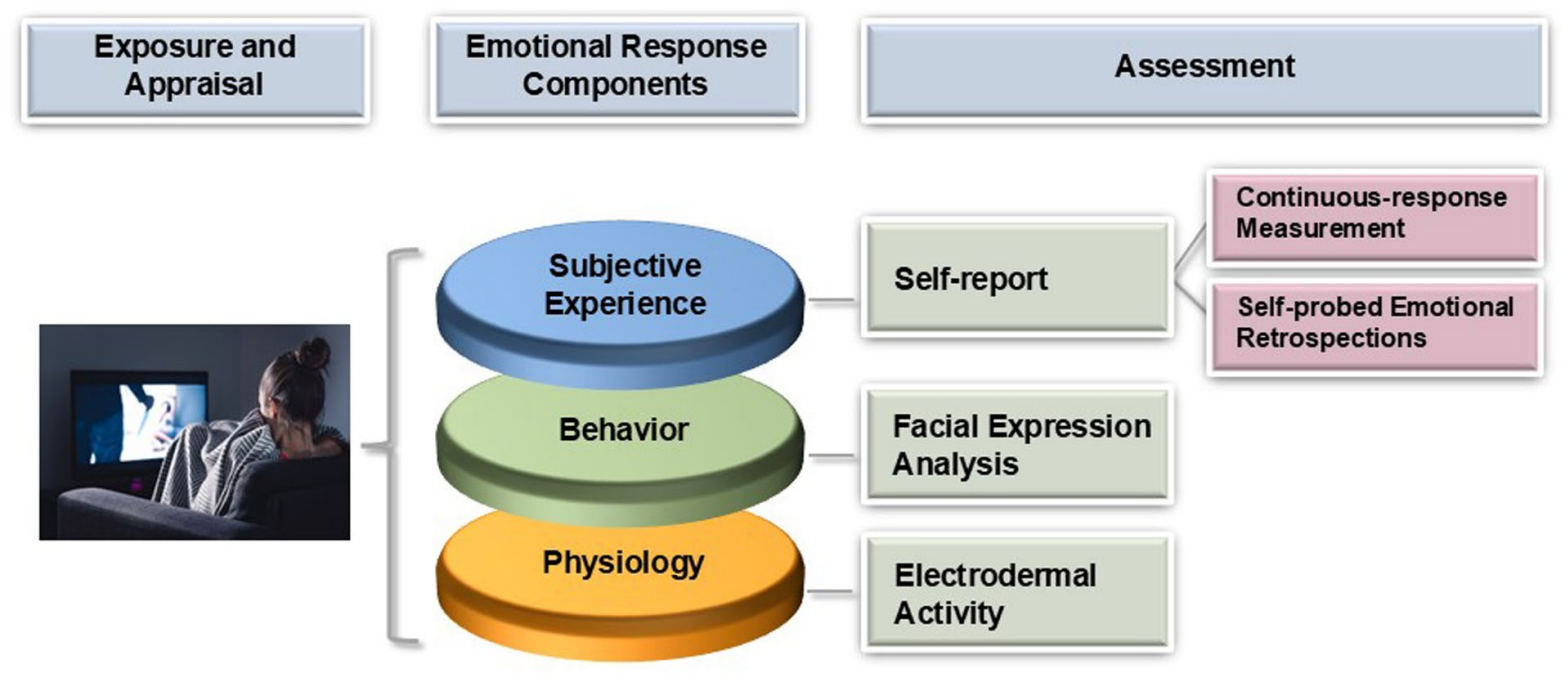
Assessment of components of dynamic emotional responses to media stimuli. Image credit: iStock.com/Tero Vesalainen.

Emotions can be defined as internal, mental states that represent valenced and conscious responses to an object (e.g., Ortony et al., 1988; Ortony, 2022). They can be distinguished from moods, which are longer-lasting diffuse affective states that are not necessarily directed toward specific stimuli (e.g., Ortony, 2022; Scherer, 2005). Most emotion theories agree that the emotional response system comprises different components: Minimal consensus conceptualizes the emotional response system as consisting of an expressive/behavioral, physiological, and experiential component (e.g., Bradley and Lang, 2002; Frijda, 2007; Lang and Davis, 2006; Mauss et al., 2024; Scherer, 2005). These components define the data sources of emotion measurement (Lang and Davis, 2006; see Figure 1). Self-report is generally viewed as a measure of subjective experience (i.e., whether people are feeling what they know as anger, sadness etc.; e.g., Ortony, 2022; Scherer, 2005; Quigley et al., 2014). Physiological measures can capture basic dimensions of emotional responses (e.g., arousal) and are essential for understanding the processes that lead to (and result from) the subjective experience of emotions (e.g., Quigley et al., 2014; Schmälzle and Grall, 2020). The analysis of facial movements constitutes a common measure of the behavioral component of emotion and helps to understand how emotional states are expressed in the face (which likely varies depending on the context and individual differences, see Barrett et al., 2019). However, researchers should not expect specific emotion categories such as anger, fear, or sadness to be identifiable by specific physiological or expressive patterns (see Behnke et al., 2022; Durán and Fernández-Dols, 2021; Lange and Zickfeld, 2021; Siegel et al., 2018). Neither physiological nor behavioral measures are direct assessments of experience (or vice versa), but rather reflect different components of emotion that all contribute to understanding the construct (Mauss and Robinson, 2009; Mauss et al., 2024). Although this review is primarily concerned with the methodological challenge of collecting emotion data rather than a discussion of emotion theory, aspects of measurement are often entangled with theory. This concerns the analysis of facial behavior in particular, which we will discuss in more detail in the corresponding section.

Self-report scales that are often used in communication research typically reflect a global and retrospective evaluation of the media experience (e.g., “How did you feel while watching this ad/browsing through these postings/listening to this story?”). These measures are an elemental part of communication research and contribute substantially to understanding various media and communication phenomena. Static measures are appropriate when using pictures or brief auditory or audiovisual stimuli that are designed to elicit a particular emotional response without substantial variability throughout the stimulus (e.g., short film clips intended to evoke a consistent emotional response). They are also useful for research in which emotional states *after* stimulus exposure are the focus of interest. However, they are unsuited to capture emotional responses as they unfold over time: The same global average score of emotional responding may represent vastly different experiences (e.g., an increase of emotional responding over time as well as a decrease, happiness when a villain succeeds, or a hero prevails). Assessing responses to dynamic media stimuli is particularly challenging because the nature of many experiences renders interruptions during reception unfeasible. For example, individuals tend to get (more or less) transported into story worlds (Gerrig, 1993; Green and Brock, 2000). Applying self-report scales during story exposure constitutes an interruption that would substantially alter the state of transportation. Thus, such a measurement would influence the experience itself in undesirable ways.

In the following sections, we present useful methodological approaches to measuring emotional responses as they occur, while maintaining minimum interruptions. We focus on electrodermal activity as a measure of physiological arousal and facial expression analysis as a behavioral measure. For the experience component of emotion, we discuss continuous response measurement and self-probed emotional retrospections.

We selected these measures because, among the available methods for obtaining continuous emotion data, they pose a relatively low threshold for researchers to incorporate into study designs.^1^ These encompass both established, reliable options and emerging methods that warrant further investigation and exploration. Among all physiological measures, electrodermal activity is a relatively accessible and easy-to-interpret indicator of sympathetic arousal. With recent advancements driven by automated analysis software, analysis of facial expressions is one of the most promising ways to obtain behavioral emotion data. Lastly, both continuous-response measurement and self-probed emotional retrospections present in-the-moment ways to measure subjective experiences that minimize interference.

It is beyond the scope of this article to discuss all possible emotion measurement methods. We do not discuss methods that primarily reflect cognitive processes (such as eye-tracking; King et al., 2019). We also do not cover physiological measures of central nervous system activity (Schmälzle and Grall, 2020). Although in the domain of psychophysiological research the neuroscience of how the brain processes naturalistic media stimuli is gaining traction and holds promise for the study of media effects (e.g., Schmälzle and Huskey, 2023; Turner et al., 2018), the equipment and training required to acquire and interpret brain data are unlikely to be available to most media and communication researchers. We do not intend to discourage researchers with the necessary training and equipment from pursuing this methodological path.

## Measuring emotions as they occur

### Psychophysiology: electrodermal activity

Emotions are embodied experiences associated with central and peripheral nervous system activity, which can be observed through psychophysiological measures (e.g., Barrett and Lindquist, 2008). Psychophysiological measures offer insights into the bodily responses associated with emotion on a moment-to-moment basis and with high temporal resolution. They can capture individuals’ immediate affective responses to stimuli, before they reach a person’s consciousness to interpret an emotional experience (Barrett and Lindquist, 2008). One of the most common and established measures of autonomic nervous system (ANS) activity is electrodermal activity (EDA). In contrast to other physiological arousal measures, EDA is considered a pure indicator of sympathetic arousal (the state of the ANS that regulates the body’s “fight or flight” response) that is unaffected by parasympathetic activity (the process regulating relaxation; e.g., Morey, 2020; Potter and Bolls, 2012). In the following, we briefly describe the general procedure of obtaining EDA data and point to relevant resources. We further discuss how EDA can be quantified, the available research to validate and test the technology, as well as opportunities and challenges to consider when integrating this methodology into media effects research.

#### Procedure

EDA is recorded via electrodes that are attached to the skin, typically the palm of the hand, the fingers, or the foot (depending on the task participants are required to do; for a comparison of different measurement sites, see van Dooren et al., 2012). Extensive technical guidelines are available for research that incorporates EDA; discussing them in detail is beyond the scope of this article. Comprehensive overviews that cover practical and technical issues like equipment, lab setup, and electrode placement, as well as the theoretical basics and interpretation of physiological measures in media research, are provided by Potter and Bolls (2012) and Dawson et al. (2017). Braithwaite et al. (2015) offer a detailed guide for data cleansing and analysis of skin conductance data. Reporting recommendations can be found in Boucsein et al. (2012).

#### What is measured

The ANS is the branch of the peripheral nervous system that plays a key role in regulating involuntary bodily functions, including emotional responses and attentional processes (Potter and Bolls, 2012). EDA is considered a pure indicator of sympathetic activation of the ANS. Sympathetic activation leads to “psychological sweating” (Potter and Bolls, 2012, p. 113), resulting in changes in the electrical conductance of the skin’s surface, which is the signal recorded by EDA measures.

Researchers should note that due to the nature of the ANS, EDA is not an exclusive indicator of affective processing. It also responds to task anticipation and performance, novelty, habituation, and deep breathing (Dawson et al., 2017). Furthermore, skin conductance offers only limited insight into emotional processing (i.e., the arousal dimension) and cannot be used to infer or differentiate between more specific emotional states (Behnke et al., 2022; Kreibig, 2010; Siegel et al., 2018). Thus, inferences on emotional responses based on skin conductance indicators must be drawn strictly within the scope of the experimental context and the materials used. They are further best combined with additional measures (e.g., self-report) to gain a fuller picture of emotional processes (e.g., Kreibig, 2010; Morey, 2020; Quigley et al., 2014).

##### Data analytic considerations

Skin conductance is most commonly quantified in terms of skin conductance level (SCL) or skin conductance responses (SCR). SCL as a tonic indicator reflects overall sympathetic activity over longer periods of time and changes only slowly. To analyze arousal based on SCL, SCL is typically averaged over any predefined time intervals that are theoretically of interest (e.g., 1 min before and after a key scene in a movie). Then, change scores are calculated based on the average SCL scores for each time interval and baseline levels (i.e., the SCL average during a short unstimulated period of time before stimulus presentation; Potter and Bolls, 2012).

SCL is affected by skin conductance responses (SCR), quick and short increases of arousal that appear as peaks in the skin conductance recording. They can be elicited by events that are unexpected, new, relevant, or aversive, but also occur as spontaneous fluctuations. In contrast to the more inert SCL indicator, SCRs can account for arousal changes within much shorter time frames (Dawson et al., 2017). To quantify arousal in terms of SCRs, the number of SCRs (peaks of at least 0.1 microSiemens within 3 s; Dawson et al., 2017) are counted within the time interval of interest. Another common approach is to calculate the mean amplitude of SCRs, that is, the average difference between onset and high point of responses (Dawson et al., 2017; Stern et al., 2001).

##### Stimuli and application contexts

EDA measures are best suited for research using audiovisual or auditory stimuli because of the time-locked presentation mode. For example, EDA measures have been used to examine dynamic emotional responses to auditory (e.g., Schmidt et al., 2023; Ellis and Simons, 2005) and audio-visual stimuli (e.g., Armstrong and Cutting, 2023; Barraza et al., 2015; Clayton et al., 2021; Han et al., 2022; Morris et al., 2019; Sukalla et al., 2016). It has also been applied in gaming research (e.g., Ravaja et al., 2008; for reviews, see Hughes and Jorda, 2021; Kivikangas et al., 2011). Studies using EDA during reading are less common (e.g., Mason et al., 2020). Synchronizing responses with a stimulus presents an additional challenge in this case due to individual differences in reading time. Using a self-paced reading paradigm allows for recording reading times for moving windows that highlight different segments of the text and that participants navigate while reading (Just et al., 1982; Keller et al., 2009). The recorded reading time can then be used to correct the EDA raw scores in each reading window.

##### Advantages

Skin conductance is a reliable and valid measure of sympathetic arousal (Koruth et al., 2015; Ravaja, 2004; van Dooren et al., 2012). Because it is not affected by parasympathetic ANS, it is easier to interpret than other physiological measures of arousal (Braithwaite et al., 2015; Dawson et al., 2017). A key benefit of skin conductance is its ability to capture involuntary processes beyond participants’ awareness and control, thus avoiding biases that typically limit self-report. Furthermore, EDA measures provide continuous, in-the-moment data that can be linked directly to the content features of the stimulus. If other continuous data exist (e.g., sentiment analyses of the stimulus), this opens possibilities for time series analyses (Ravaja, 2004; Suckfüll, 2010). Recent developments in wearables also enable research outside the laboratory, reducing barriers to incorporating psychophysiological measures into study designs and increasing the external validity of EDA-based research. However, not all wearables perform the same. Researchers should check whether the device has been validated with more established laboratory systems (Ferreira et al., 2023; Konstantinou et al., 2020; van der Mee et al., 2021).

##### Limitations

Although measuring EDA is relatively inexpensive and straightforward compared with other psychophysiological measures, conducting physiological research in general is more costly, time-intensive, and requires an extensive lab setup compared with simpler methods such as self-report. Further, additional training is required to obtain, process, and interpret data successfully. Inspecting data and dealing with common issues like signal loss, artifacts (e.g., drift in the SCL, motion artifacts; Braithwaite et al., 2015), or non-responders (an estimated 10 % of the population do not show any variation in skin conductance; Dawson et al., 2017) can be time-consuming, especially for researchers who are new to physiological research. Because of this increased effort, the sample sizes that can be achieved with physiological research are typically limited.

### Behavior: automated facial expression analysis

Emotional states often lead to observable facial muscle contractions. Proponents of a basic emotion approach have suggested that emotional states can be identified based on specific facial muscle configurations (Cordaro et al., 2018; Ekman, 1993; Keltner et al., 2019). Although the hypothesis of prototypical facial expressions related to emotional experiences is highly controversial, emotion scholars agree that emotional states often lead to facial behavior (Barrett et al., 2019). Therefore, analyzing the muscle contractions accompanying emotional experiences is meaningful, irrespective of the theoretical background. The development of computer-vision machine learning technology in recent years has made it possible to automate this otherwise laborious process and generate intensive facial-behavior data during media reception (Küntzler et al., 2021; Stöckli et al., 2018). In the following, we discuss the procedure of conducting automated facial expression analysis (FEA), the available research to validate and test the technology, and the opportunities and challenges of integrating this methodology into media effects research.

#### Procedure

The most prominent and widely used framework to describe facial muscle contractions is the Facial Action Coding System (FACS) (Cohn and Ekman, 2005; Ekman and Friesen, 1978). The FACS consists of 46 action units (AUs) that identify different facial movements (e.g., raising of the upper lip, wrinkling of the nose). The coding of AUs is typically performed based on video material of participants, frame by frame. Different software applications have been developed in recent years to automate this process, for example, FaceReader (by Noldus) and FACET (by iMotions; for a performance comparison of different software, see Küntzler et al., 2021; Stöckli et al., 2018). Facial expressions are typically recorded in front of a screen, which enables a full-frontal view of participants’ faces. To ensure high data quality, the participant’s face should be well lit, and ideally, a neutral background should be chosen. Covering parts of the face (e.g., by facial hair, glasses, excessive make-up, or by participants touching their faces) may impair data quality (Küntzler et al., 2021). To account for this, researchers should have necessary accessories available in the lab (e.g., hair pins to remove fringes), instruct participants to come to the lab prepared (e.g., by asking them to wear contact lenses and to shave or trim beards, if possible), and to keep their hands out of their faces.

#### What is measured

Research has found that FEA software is generally able to differentiate intensities of AUs and standardized prototypical facial expressions of emotions (Beringer et al., 2019; Calvo et al., 2018; Höfling et al., 2022; Lewinski, 2015; Lewinski et al., 2014; Stöckli et al., 2018). However, naturally occurring expressions are often more nuanced and complex. Detection accuracy in these instances tends to be lower compared with prompted and standardized portrayals (Höfling et al., 2020; Küntzler et al., 2021; Stöckli et al., 2018), whereas humans can recognize more subtle and complex emotional expressions (Yitzhak et al., 2017). Of note, the detection accuracy of FEA software varies for different emotions: Expressions of happiness/joy is recognized most accurately. Accuracies for negative emotions, such as anger, fear, and sadness, are lower (Calvo et al., 2018; Lewinski et al., 2014), particularly for non-standardized and naturally occurring expressions (Küntzler et al., 2021; Stöckli et al., 2018; Zempelin et al., 2021). When considering the valence and arousal dimensions of emotions, the sensitivity of FEA software to differentiate between observed pleasant and neutral responses is comparable to that of facial electromyography (EMG), which records the electric potential of facial muscles associated with positive and negative valence. However, it is less sensitive in differentiating unpleasant and neutral responses and does not seem suited for measuring emotional arousal (Höfling et al., 2020).

Although the aim of this review is to highlight different measurement options while remaining neutral in terms of theory, it should be noted that the classification of emotion categories in all FEA applications builds on basic emotion theory. In particular, the view of emotions as biologically basic is disputed in the literature (for a review, see Barrett et al., 2019). Most emotion scholars agree that emotional states often lead to facial behavior and that this should be taken into account in a comprehensive assessment of emotions. It is also uncontroversial that people use basic emotion terms (e.g., anger, joy, sadness) to organize their knowledge about and describe their experiences of emotions (Barrett et al., 2019; Ortony, 2022). However, evidence suggests that facial expressions associated with experienced emotions are likely more heterogeneous and less prototypical than proponents of biologically basic emotions suggest (for meta-analyses, see Durán and Fernández-Dols, 2021; Reisenzein et al., 2013; see Mauss et al., 2024 for a general discussion of emotional response system coherence). One exception is amusement, which highly co-occurs with laughter and smiling (Durán and Fernández-Dols, 2021). Conversely, facial movements do not necessarily signal an emotional state and are influenced by context (Barrett et al., 2019). For example, facial expressions classified as angry may result from a genuine experience of frustration, but could also reflect cognitive effort or concentration associated with a task (e.g., Talen and den Uyl, 2022; Yu and Ko, 2017).

#### Data analytic considerations

Automated FEA yields continuous data for various emotion categories. Most FEA software applications also allow the analysis of AUs rather than emotion categories, which is relevant for researchers who wish to conduct FEA without the background of basic emotion theory. Analyzing AUs rather than relying on the categorization of emotional expressions has been encouraged by some emotion scholars. Conducting FEA in this manner may require a more descriptive approach because an alternative theoretical framework that allows valid and reliable inferences of emotional expressions from facial behavior is currently not available. Barrett et al. (2019) recommends combining FEA with self-report and to consider the context in which facial movements occur (see also Mauss et al., 2024).

#### Stimuli and application contexts

As with psychophysiological data, FEA software is best suited for measuring responses to audiovisual and auditory media stimuli, as this allows to synchronize audience responses with the stimulus. However, applications using text-based stimuli are also feasible. Measuring facial movements using FAE software is possible in and outside of laboratory settings (most providers offer online applications), provided that researchers ensure proper conditions to maximize the likelihood of accurate expression classification (see Küntzler et al., 2021).

As a rather novel technology, research using automated FEA to infer emotional responses during media use is scarce. However, some research has applied this method successfully to study continuous emotional responses to music (Weth et al., 2015), in a user experience setting (Talen and den Uyl, 2022), to examine the role of emotional responses for the persuasive effects of a movie (Appel et al., 2019) and for sharing of advertisements (McDuff and Berger, 2020). Importantly, these studies also provide evidence of the predictive validity of emotion data gathered using FEA software (Appel et al., 2019; McDuff and Berger, 2020).

#### Advantages

Whereas much of the FACS research has traditionally relied on intensively trained human coders, the manual coding of continuous responses to dynamic media stimuli presents a colossal task (Barrett et al., 2019; Stöckli et al., 2018). Thus, automated FEA is a promising method to generate intensive datasets of facial behavior during media reception, which cannot be achieved using manual coding. Automated FEA software has been found to perform similarly to human coders (Calvo et al., 2018; Küntzler et al., 2021; Lewinski et al., 2014). These applications are continuously under development; thus, their performance can be expected to improve over time.

Compared with facial EMG, automated facial expression analysis (FEA) enables easier data collection, is less intrusive for participants (because no electrodes need to be attached in their faces, possibly obstructing their view and causing an artificial situation), and has a low risk for motion artifacts. In addition, facial expression data are free from biases (e.g., social desirability, memory biases, demand characteristics) that may affect self-report (e.g., Quigley et al., 2014).

#### Limitations

At this point, automated FEA is less accurate and sensitive for classifying negative emotional expressions than positive expressions (e.g., Calvo et al., 2018; Höfling et al., 2020; Küntzler et al., 2021; Zempelin et al., 2021). This likely boils down to the fact that the categorization of facial expressions relies on basic emotion theory, which has been increasingly criticized by emotion scholars. Because an alternative framework for categorizing constellations of AUs in terms of emotion expression is lacking, researchers who wish to study facial expressions without the lens of basic emotion theory are left with an inductive approach to data analysis (for a detailed discussion and recommendations, see Barrett et al., 2019).

### Self-report measures

Given their easy use and relatively straightforward interpretation, self-report measures are the most common way to measure emotions. In contrast to physiological measures and observations of facial behavior, self-reports allow for more direct inferences of emotional experiences and offer insight into the subjective feeling component of emotions (Barrett et al., 2007; Scherer, 2005).

Several limitations are inherent to all self-report measures. For one, they depend on an individual’s consciousness of an experience and their ability and willingness to report it (e.g., Nisbett and Wilson, 1977; Quigley et al., 2014; Scherer, 2005). Furthermore, self-reports can be influenced by demand effects and social desirability biases, such that participants’ response behavior might be affected by their assumptions of what the study is about or by perceived social norms (Moulds et al., 2023).

More specific challenges arise when using self-report to capture dynamic emotional responses to media stimuli. Simply probing emotional experiences mid-exposure by pausing the stimulus or retrospective self-reports based on predefined cues (e.g., film stills, event descriptions) might be sufficient for some research purposes, but can cause problems in terms of reactivity and disrupt relevant processes (e.g., Durkin and Wakefield, 2008; Kalch and Bilandzic, 2017; Larsen and Seilman, 1988). Furthermore, both options do not capture potentially relevant variance in emotional responses outside of these predefined measurement points. In the following, we discuss methods that avoid or mitigate these issues.

#### Continuous response measurement

Continuous response measurement (CRM) or real-time response measurement (RTR) describes measurement systems that allow participants to provide continuous ratings of subjective experiences while being exposed to a stimulus (Biocca et al., 1994; Maier et al., 2009). In principle, CRM is suited for measuring any aspect of subjective emotional experience that participants can indicate on up to two dimensions simultaneously.

#### Procedure

CRM is administered using devices such as handheld rating dials, sliders, joysticks, or via web interfaces on touchscreens of smartphones and tablets (Wagner et al., 2021). Participants use these tools to provide continuous feedback on up to two rating dimensions in response to a media stimulus. Tools and systems to implement CRM measurement are widely available, such as the Software for Continuous Affect Rating and Media Annotation (CARMA) (Girard, 2014), the Software for Dual Axis Rating and Media Annotation (DARMA) (Girard and Wright, 2018), EMuJoy (Nagel et al., 2007), or the emoTouch web application (Louven et al., 2022).

#### What is measured

Most CRM studies use a single dimension to assess subjective emotional experiences, typically a bipolar valence scale (e.g., Siegenthaler et al., 2021; Winkler et al., 2022) or a unipolar scale of a specific emotion category, such as amusement or sadness (e.g., Mauss et al., 2005). Some studies have also measured more complex affective experiences such as suspense (e.g., Bente et al., 2022). After a short learning period to get accustomed to the procedure, participants typically perceive continuous ratings on a single dimension as a manageable task (Ruef and Levenson, 2007; Wagner et al., 2021). Importantly, research has found the use of a single-item RTR measure to be non-reactive, meaning that the task itself does not seem to alter subjective experiences (Hutcherson et al., 2005; Mauss et al., 2005; Wagner et al., 2021).

Some studies measured subjective experiences on two dimensions simultaneously, either by using two independent sliders (Fayn et al., 2022; Lottridge and Chignell, 2010) or by navigating a joystick to indicate responses within a two-dimensional grid (e.g., positive and negative valence; Larsen et al., 2009). This allows for the examination of mixed subjective emotional experiences (i.e., positive and negative emotions at the same time), which cannot be adequately represented on a single bipolar scale. Few studies have examined the cognitive load associated with two-dimensional RTR measures and possible issues related to reactivity. The available evidence suggests that although cognitive load and perceived difficulty increase with two-dimensional measures, participants can become accustomed to the task (Fayn et al., 2022; Lottridge and Chignell, 2010). The use of two independent sliders appears to be easier for participants and reduces measurement-related dependencies compared to a two-dimensional grid (Fayn et al., 2022). However, more research is needed to determine the conditions under which two-dimensional CRM of subjective emotional experiences can be applied successfully. Rather than measuring two dimensions simultaneously, researchers can assess multiple dimensions of subjective experience by exposing participants to a stimulus repeatedly and instructing them to indicate how they remember feeling during the first time of exposure. Mauss et al. (2005) found subjective emotional experiences (sadness and amusement) measured through this method of stimulated recall upon repeated viewing of a film to correlate strongly with previous continuous ratings.

#### Data analytic considerations

CRM yields continuous data of the respective measurement dimensions.

#### Stimuli and application contexts

CRM is best suited to audiovisual and auditory stimuli. It has been used in various areas of research, such as political communication studies on audience evaluations of political speeches or televised debates (e.g., Boussalis and Coan, 2021; Friederike Nagel et al., 2012), research on affective and cognitive processing of narratives and entertainment (e.g., Armstrong and Cutting, 2023; Bente et al., 2022; Tchernev, 2022; Tchernev et al., 2023; Winkler et al., 2022), health communication (e.g., Siegenthaler et al., 2021), or research on music and aesthetic experiences in response to art (e.g., Svanås-Hoh et al., 2022; Wagner et al., 2021). In principle, the ubiquity of personal smartphones presents an opportunity for applications outside of the lab, and some tools for tablets and smartphones are available to facilitate CRM studies in naturalistic settings (e.g., the free web application emoTouch; Louven et al., 2022). However, research utilizing this potential remains limited (Boydstun et al., 2014; Maier et al., 2016; for a scoping review of communication studies, see Schnauber-Stockmann and Karnowski, 2020).

#### Advantages

A major advantage of this method is that it allows for an inexpensive and straightforward way to collect high-resolution real-time subjective experience data while avoiding the memory biases often associated with retrospective self-reports. Furthermore, it can also be implemented outside the lab in naturalistic settings or online studies (e.g., Maier et al., 2016).

#### Limitations

The main limitation of CRM is that it typically allows the measurement of only one or two dimensions of experience. Researchers interested in a broader assessment of emotional experience must rely on workarounds that come with their own limitations (e.g., repeated exposure, between subject variation of experience dimensions).

#### Self-probed emotional retrospection

Self-probed emotional retrospection is a method to capture rich subjective emotional experiences of text-based stimuli as they occur within the reader. Larsen and Seilman (1988; Seilman and Larsen, 1989) first used a similar method that united the advantages of probing and thinking aloud to study personal memories evoked by literature. It was later adapted by Eng (2002; see also Mar et al., 2011 for a discussion) to examine the role of emotional experiences, memory, and distracting thoughts in reading comprehension.

#### Procedure

The method comprises two steps. Participants are instructed to mark an “E” (for emotion) on the margin of a text whenever they experience an emotion while reading. After they finish reading, participants are asked to return to these markings and specify their emotional experiences for each “E,” for example, by rating the experienced intensity of different emotion categories. For lengthy stimuli, researchers are recommended to limit the amount of “E”s to be qualified after reading (but not the amount of “E”s to be marked during reading). Interindividual differences in the number of “E”s people mark can be quite large, thus, researchers may instruct participants to only select the most relevant markings to specify (see Winkler et al., 2023).

#### What is measured

Because this method does not rely on predefined measurement points, it is particularly suited to capture highly idiosyncratic subjective emotional experiences (e.g., emotional memories, which cannot be anticipated from the content of the stimulus). Studies using lengthy and naturalistic stimuli likely benefit using this method the most. In principle, any self-report scale to assess the emotional experiences for each “E” can be combined with this method depending on the researcher’s needs (e.g., the Circumplex Model of Affect, Posner et al., 2005; the Modified Differential Affect Scale, Renaud and Unz, 2006; the Geneva Emotion Wheel, Scherer, 2005; Scherer et al., 2013). However, brief scales are advisable to prevent fatigue from completing long scales repeatedly.

Initial data exists that examines this method’s validity, accuracy, and non-reactivity. Participants in Larsen and Seilman (1988) found the task of marking parts of a text while reading to be easy and non-intrusive. Furthermore, participants rarely forgot what they had been reminded of when using self-probed retrospection (although unfortunately the study did not include a control group). Some evidence underscores the method’s sensitivity to capture subjective emotional experiences of varying quality (Koopman 2016) and intensity (Winkler et al., 2023). Koopman (2016) found that the method was able to capture variance in aesthetic and ambivalent emotional experiences resulting from stylistic variations in stories. Winkler et al. (2023) showed that a greater amount of “E”s marked was predicted by individuals’ tendencies to seek out emotional experiences (need for affect). Furthermore, subjective emotional experiences measured in this way were associated with theoretically related processes (transportation) and outcomes (social sharing intentions; Winkler et al., 2023). Clearly, more research is needed to examine whether this method holds advantages over free retrospective recall in terms of memory biases and whether it is reactive.

#### Data analytic considerations

Because the frequency and spacing of observations (e-markings) varies between individuals, the data generated through self-probed emotional retrospection is not fully continuous. Depending on how researchers intend to analyze the data, two data structures are possible: The variables in the dataset reflect the e-markings by the participant in their chronological order. This implies that individual observations are not comparable between participants (i.e., “E1” may refer to different events in the narrative for different individuals). This option is suitable if an aggregate indicator of subjective emotional changes is computed (e.g., Winkler et al., 2023).

Alternatively, variables in the dataset may also reflect properties of the stimulus (e.g., a paragraph, a line in the text). Observations (e-markings) are allocated to specific units of analysis (e.g., if “E1” appears in line 6 of the text, experience ratings given for “E1” are entered for the variable reflecting the line 6). The remaining data points may be treated as missing values or imputed, depending on the type of data analysis planned (e.g., in contrast to time-series analyses, multilevel modeling can accommodate an unequal number of observations per person; Snijders and Bosker, 2012). This option is appropriate for researchers who plan to analyze the data in conjunction with the text and/or intend to conduct between-participant comparisons.

#### Stimuli and application contexts

Whereas the methods discussed so far are most readily applicable to audiovisual or auditory stimuli, self-probed emotional retrospection presents a feasible way of obtaining rich data on subjective emotional experiences while reading text-based stimuli. It requires no additional methods or workarounds (i.e., eye-tracking, reading windows) if the goal is to match emotional experiences to the text. Only a few studies have used this method so far. Koopman (2016) applied it to investigate the effects of foregrounding on the emotional experience of a novel, and Winkler et al. (2023) used it to quantify the experience of emotional shifts in narrative persuasion.

In instances in which CRM is not feasible and/or a more comprehensive assessment of subjective experiences is desired, the method could also be adapted to contexts other than literature. We discuss possible examples in the discussion section.

#### Advantages

This method combines the advantages of thinking aloud and retrospective probing. It records subjective emotional experiences in response to a text in the moment they occur, but without disturbing the reading experience by prompting participants to report on their experiences immediately, which could possibly prompt an analytic reading mode (Larsen and Seilman, 1988). Compared with CRM, this method allows for a richer assessment of feelings and enables researchers to study the dynamics of emotionally complex experiences, including the occurrence of mixed emotional experiences. Because the measurement points are determined by the reader, the demand characteristics of this method should be lower than those of researcher-induced probing, although research is needed to investigate this hypothesis.

#### Limitations

The downside of this is approach is that self-probed emotional retrospection yields a lower level of resolution than other methods discussed so far, and measurement points are not directly comparable between participants. Furthermore, although the marking of subjectively emotion-eliciting passages in the text creates an anchor that should later help to cue recall more accurately, it is still possible that the retrospective nature of qualifying emotional experiences prompts a higher degree of rationalizing and memory bias than CRM. Further research is needed to investigate this.

## Discussion

### Contributions to key areas of media psychology and communication research

Many media stimuli and related interactions are dynamic. Thus, we argue that empirical approaches to measuring emotional states should reflect dynamic changes in affective responses. In the following section, we support this claim: How can the dynamic measurement of emotional processes contribute to key areas of communication and media effects research? We highlight some examples and discuss how dynamic measures of emotion can be applied and adapted to answer relevant open questions in central areas of investigation.

#### Narrative effects research

The dynamic nature of narrative structures—characterized by a setting of the stage, events that move the story forward and a rise and fall of action as a protagonist faces conflicts and challenges—have been described by narrative theory since Aristotle (1994; e.g., Freytag, 1905). Research using large-scale language analysis has recently confirmed these structural features in a variety of narrative formats (e.g., books, movies, but also science communication, or TED talks; Boyd et al., 2020). Sentiment analyses have further revealed typical emotional trajectories that are prevalent in movies (e.g., del Vecchio et al., 2021; Dale et al., 2023) and novels (Reagan et al., 2016).

The dynamic emotional responses audiences experience in response to a narrative’s unfolding events are suggested to be driving forces behind narrative persuasion (Nabi, 2015; Nabi and Green, 2015; Winkler et al., 2023). This proposition has generated research efforts in the domain of health communication in particular (e.g., Adams et al., 2022; Alam and So, 2020; Fitzgerald et al., 2020; Ophir et al., 2021; Ort et al., 2023; Shen and Li, 2023; Siegenthaler et al., 2021). However, these studies rarely include continuous measures of emotional responses throughout the stimulus to investigate emotional responses as mechanisms of effects (but see Siegenthaler et al., 2021). To advance research in the domain of health communication and narrative persuasion, research needs to move beyond using post-exposure self-report scales to measure emotional processes during media reception. Instead, the dynamic nature of emotional responses should be reflected methodologically. This allows to investigate hypotheses related to emotional responses to specific elements in a narrative as mechanisms of narrative impact (e.g., Appel et al., 2019; Armstrong and Cutting, 2023). For example, Dahlstrom (2010, 2012) demonstrated that information placed at locations with causal relevance to the narrative leads to higher information acceptance. Similarly, the nature of emotional responses (e.g., in terms of their intensity or valence) during these causally relevant narrative events may be key to understanding narrative effects.

#### Social media research

An enormous body of research has investigated how social media use affects various well-being and ill-being parameters, which has been synthesized in multiple meta-analyses (for umbrella reviews, see Appel et al., 2020; Valkenburg, 2022). The results of these meta-analyses typically point toward weak and heterogenous effects and suggest that influences of social media are not adequately described in terms of averaged between-person effects (Valkenburg, 2022). Rather, experiences during social media use vary depending on individual differences and motivations of social media users (Beyens et al., 2021).

Emotions likely influence how social media use affects well-being and ill-being. For example, social media offers plenty of opportunities for (mostly upward) social comparison (McComb et al., 2023). The emotional effects of upward social comparison can be described in terms of envy (with negative implications for well-being), but also inspiration (Meier et al., 2020). Emotions can be understood as both causes and effects of social media use and are likely shaped by individual and contextual differences. Which individual and contextual factors shape the positive and negative emotional effects of upward social comparisons? How does this affect users’ further activities and outcomes in terms of well-being? Within-person perspectives using continuous emotion measures can help understand the dynamic interplay between these variables.

The methods discussed here can advance social media research in both naturalistic and experimental settings (see also Griffioen et al., 2020). For example, the advancement of wearable technology enables the assessment of physiological measures during real-life social media behavior. To capture emotional experiences, a version of self-probed emotional retrospection could be applied, for example, by asking participants to take screenshots of social media content and interactions that subjectively were of emotional relevance to them. These screenshots can then serve as cues for participants to describe their emotional experiences later (i.e., after a social media browsing session) in a media diary or survey. The screenshots can also be analyzed for their emotion-eliciting properties through content analysis (provided identifying information is removed) or by asking participants to describe the object of their emotional experience in each instance. In laboratory settings, the possibility of adapting CRM to the requirements posed by the activity of using social media might also be explored. For example, participants may be able to indicate emotional experiences using a foot pedal instead of a handheld device, although this possibility needs to be tested for feasibility and possible reactivity.

#### Virtual reality research

Another area of interest is user experiences in virtual worlds. Virtual reality (VR) can take immersive experiences to the next level by allowing users to embody and act as characters while being present in a virtual world. VR can elicit strong and complex emotions such as awe or inspiration (Kahn and Cargile, 2021; Quesnel and Riecke, 2018). At the same time, interactive immersive settings like VR are demanding in various ways (cognitive, emotional, physical, social, Bowman et al., 2021), which affects the allocation of users’ limited cognitive resources and can lead to potentially detrimental outcomes (e.g., cybersickness; Breves and Stein, 2023). For these reasons, VR has sparked the interest of researchers interested in understanding entertainment experiences in VR as well as the potential prosocial effects of this technology (e.g., improving attitudes toward minority groups, improving climate change related attitudes and behavior; Kahn and Cargile, 2021; Leung et al., 2022). Understanding how the demands posed by interacting in virtual worlds shape emotions and how these, in turn, mediate effects on outcomes such as persuasion, social cognition, and entertainment poses an avenue for future research.

Physiological measures are an obvious choice for assessing physiological processes during interactions in VR (see for example Zhang et al., 2024, for an investigation of arousal escalation and decay in VR). Capturing facial behavior presents a greater challenge, given that the head mounted display covers a large part of the participant’s face. However, technological solutions potentially enabling FEA in VR settings are currently being developed and may be feasible in the future (Chen and Chen, 2022). When assessing subjective emotional experiences during VR sessions, it is important to select a method that requires the lowest possible cognitive demand for participants to execute. An adapted version of self-probed emotional retrospection might be feasible. For example, time stamps of significant emotional experiences can be recorded by using a clicking device. Alternatively, time stamps based on verbal cues by the participant could be recorded by the experimenter. These time stamps can then be used to revisit the corresponding moments in the VR stimulus and ask participants to qualify their subjective emotional experiences during that point.

### Recommendations for future research

Based on the eminent literature as well as our own experience in the field of continuous emotion measurement, we would like to offer some methodological and practical recommendations for future media effects research that intend to include process measures of emotion during media reception.

1. Measures of different emotion components should not be assumed to be interchangeable.

When combining different emotion measures, researchers should not expect a one-to-one relationship between these different data sources (see Mauss et al., 2024). Researchers should keep in mind the meaning of different emotion measures and the inferences they warrant. This involves carefully considering which methods align best with the research aims when planning a study. For example, if arousal is relevant to the research question, a physiological measure should be used, especially if more subtle arousal changes are of interest. Research suggests that people can misjudge their physiological arousal (Sze et al., 2010), and especially in the case of CRM, self-reporting arousal throughout a media experience might be rather demanding (Lottridge and Chignell, 2010). Conversely, if researchers seek insight into the experience of emotions (i.e., feelings), a self-report measure is required. Neither psychophysiological nor behavioral data (e.g., facial expressions) are suitable to infer subjective emotional experiences (e.g., Barrett, 2016; Behnke et al., 2022; Durán and Fernández-Dols, 2021; Siegel et al., 2018). Self-report remains the most direct measure of the subjective feeling component of emotion (Barrett, 2016; Scherer, 2009). However, self-report comes with certain limitations, which physiological and behavioral measures are not affected by. For example, certain mental processes are not accessible through introspection (Nisbett and Wilson, 1977) and the influence of social desirability may deter participants from disclosing their feelings, whereas physiological measures can reveal affective processes beyond an individual’s conscious control (and are thus often referred to as objective, in contrast to the subjectiveness of self-report measures). Thus, incorporating emotion data from various sources and capturing the physiological, experiential, and behavioral components allows for a more comprehensive assessment of emotional responses (e.g., Bradley and Lang, 2002). Multi-method studies can help to understand how different components of the emotional response system and their interactions shape media effects and therefore present a particularly valuable approach.

2. Interpretation of distinct emotion categories should be aided by self-report.

Empirical evidence to date does not support the notion of “affect programs” in a sense of specific elicitation patterns of ANS activity or facial expressions (except for amusement) that allow to clearly differentiate between distinct emotion categories (Durán and Fernandez-Dols, 2021; Siegel et al., 2018). Thus, inferences about specific emotion categories (e.g., anger, guilt, hope, nostalgia) are limited when based solely on physiological and facial expression data. If researchers intend to investigate specific emotion categories rather than—or in addition to—the broader valence and arousal dimensions of emotion, self-report measures should be included.

If a continuous self-report measure is unfeasible in a specific context (e.g., too demanding for participants, incompatible with the task or other measures), a retrospective assessment is an appropriate compromise. Cues can help participants recall their experiences. For FEA, one possibility is to use live analyses of facial expressions (i.e., expressions are analyzed by the software in the moment they are recorded, an option that is offered by some providers). Time stamps of high-intensity emotional expressions may be recorded and later replayed to participants, who could then describe their experience of the moment in question (Barrett et al., 2019).

3. When using physiological measures for the first time, collaborate with researchers who have experience with these methods.

Researchers should be aware that successfully incorporating psychophysiological measurements into their studies requires considerably more effort, time, and additional training compared to other methods, and researchers should expect things to go wrong at first. Therefore, researchers are well advised to collaborate with or seek advice from others who are experienced in the domain of psychophysiological measures. Furthermore, technical support should also be ensured to avoid technical issues that could cause delay in research projects.

4. Define an *a priori* analysis plan.

Assessing multiple continuous emotion measures creates large amounts of data. Analyzing these data presents a complex task. It is important to articulate clear questions and expectations that guide data analysis and interpretation, even when conducting exploratory research. This pertains to both the selection of emotion measures for inclusion in a study and the appropriate data analysis methods to avoid searching for the needle in a haystack. A theory-guided assessment is crucial in this regard. Whenever possible, researchers should develop expectations of emotional responses to media stimuli based on theory. Furthermore, multi-method studies that combine different emotion measures should have a foundation in emotion theory that helps to integrate these different data sources successfully. Lange and Zickfeld (2021) suggested a network analysis approach for this purpose, which is built on the notion that emotions are distinct constructs with fuzzy boundaries, meaning that they share connections in their components.

Recently, communication and media effects research has made considerable progress conceptually articulating the change processes of communication phenomena. Brinberg and Lydon-Staley (2023) described nine ways in which change can be described (e.g., growth/decay, entrainment, ruptures) and recommended analytic techniques suitable for modeling these change processes. For example, the question of whether physiological arousal produces more intense subjective experiences could be analyzed as a process of entrainment. Shen and Li (2023) described the fluctuations of subjective fear and hope in response to a threat appeal in terms of a U-shaped (or inverted U-shaped) curve, which may be quantified using latent growth curve modeling. How emotional changes are conceptualized and analyzed matters tremendously for the outcomes that can be predicted by these emotional dynamics. For example, Shen and Coles (2015) demonstrated that a linear within-person increase in subjective fear in response to a fear appeal message predicts reactance, whereas a reverse U-shaped pattern of fear experience (increase, then decrease) predicts the persuasive effects of the message.

5. The data analytic strategy should appropriately reflect the change processes of interest.

Continuous measures present the opportunity to sample dynamic changes in emotional processes with high temporal resolution. Ideally, researchers should opt for analytic strategies that reflect the dynamic and longitudinal nature of their data because aggregate (static) measures cannot capture the within-person changes occurring in response to media stimuli adequately (e.g., Koruth et al., 2015). The appropriate data analytic strategy is determined by various factors. This includes the amount of data points available, the change processes of interest, the model specifications (i.e., whether there are moderators or mediators to consider), whether there are missing data or a different number of observations between participants (which multi-level modeling approaches can accommodate, but time-series analyses or structural equation modeling cannot; Snijders and Bosker, 2012). Brinberg and Lydon-Staley (2023) offer a comprehensive overview of appropriate modeling strategies for various change processes.

## Conclusion

This article addresses one of the main methodological challenges of communication and media effects research by discussing ways to measure emotional responses to media in the moment they occur. Understanding how emotional dynamics during media reception unfold can help advance various domains of media effects research. We spotlighted methodological approaches that, compared to other continuous emotion measures, offer a low threshold for integration into future media reception and effects studies. These approaches present a diverse toolkit that is suited to capture aspects of the physiological, expressive, and experiential components of emotion: Electrodermal activity, automated facial expression analysis, continuous response measurement, and self-probed emotional retrospections. These methods can be flexibly adapted to various research contexts. Furthermore, technological advances (e.g., wearables, the ubiquity of smartphones) have made it easier than ever to apply methods that were previously confined to a laboratory context to naturalistic settings. With this article, we hope to help researchers be aware of the challenges, but also recognize the potential of measuring emotional processes. We thereby hope to encourage future studies to empirically account for the dynamic nature of emotional processing of media content by incorporating continuous measures of emotion.

## Footnotes

^1^We acknowledge that in some underfunded academic systems what we describe as a low threshold can represent a higher barrier. Financial limitations may affect the feasibility of incorporating certain emotion measures (particularly psychophysiological measures or facial expression analysis). However, the self-report measures discussed here represent rather cost-effective options.
